# Increasing on-treatment hepatocellular carcinoma risk with decreasing baseline viral load in HBeAg-positive chronic hepatitis B

**DOI:** 10.1172/JCI154833

**Published:** 2022-05-16

**Authors:** Won-Mook Choi, Gi-Ae Kim, Jonggi Choi, Seungbong Han, Young-Suk Lim

**Affiliations:** 1 Department of Gastroenterology, Liver Center, Asan Medical Center, University of Ulsan College of Medicine, Seoul, South Korea.; 2 Department of Internal Medicine, Kyung Hee University School of Medicine, Seoul, South Korea.; 3 Department of Biostatistics, Korea University College of Medicine, Seoul, South Korea.

**Keywords:** Hepatology, Virology, Hepatitis, Liver cancer

## Abstract

**BACKGROUND:**

It is unclear whether the level of serum hepatitis B virus (HBV) DNA at baseline affects the on-treatment risk of hepatocellular carcinoma (HCC) in hepatitis B e antigen–positive (HBeAg-positive), noncirrhotic patients with chronic hepatitis B (CHB).

**METHODS:**

We conducted a multicenter cohort study including 2073 entecavir- or tenofovir-treated, HBeAg-positive, noncirrhotic adult CHB patients with baseline HBV DNA levels of 5.00 log_10_ IU/mL or higher at 3 centers in South Korea between January 2007 and December 2016. We evaluated the on-treatment incidence rate of HCC according to baseline HBV DNA levels.

**RESULTS:**

During a median 5.7 years of continuous antiviral treatment, 47 patients developed HCC (0.39 per 100 person-years). By Kaplan-Meier analysis, the risk of HCC was lowest in patients with baseline HBV DNA levels of 8.00 log_10_ IU/mL or higher, increased incrementally with decreasing viral load, and was highest in those with HBV DNA levels of 5.00–5.99 log_10_ IU/mL (*P* < 0.001). By multivariable analysis, the baseline HBV DNA level was an independent factor that was inversely associated with HCC risk. Compared with HBV DNA levels of 8.00 log_10_ IU/mL or higher, the adjusted HRs for HCC risk with HBV DNA levels of 7.00–7.99 log_10_ IU/mL, 6.00–6.99 log_10_ IU/mL, or 5.00–5.99 log_10_ IU/mL were 2.48 (*P* = 0.03), 3.69 (*P* = 0.002), and 6.10 (*P* < 0.001), respectively.

**CONCLUSION:**

On-treatment HCC risk increased incrementally with decreasing baseline HBV DNA levels in the range of 5.00 log_10_ IU/mL or higher in HBeAg-positive, noncirrhotic adult patients with CHB. Early initiation of antiviral treatment when the viral load is high (≥8.00 log_10_ IU/mL) may maintain the lowest risk of HCC for those patients.

**FUNDING:**

Patient-Centered Clinical Research Coordinating Center (PACEN) (grant no. HC20C0062) of the National Evidence-based Healthcare Collaborating Agency; National R&D Program for Cancer Control through the National Cancer Center (grant no. HA21C0110), Ministry of Health and Welfare, South Korea.

## Graphical abstract

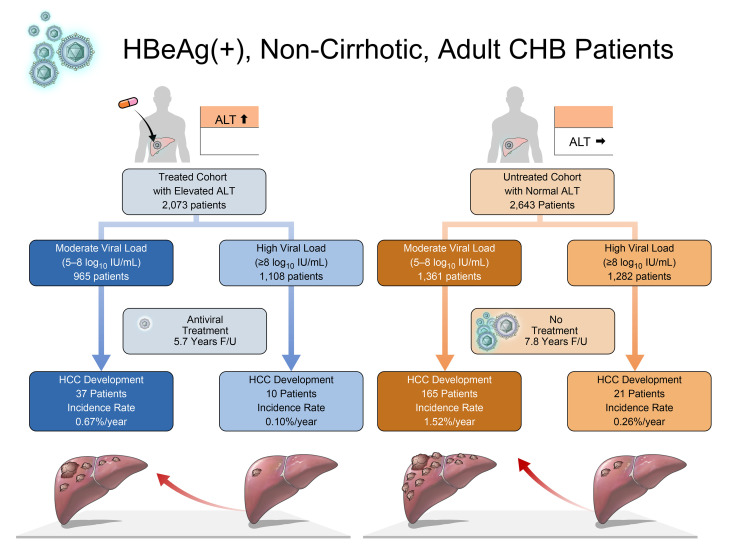

## Introduction

Hepatocellular carcinoma (HCC), the most common form of primary liver cancer, is the third leading cause of cancer-related mortality worldwide (1). Global deaths from HCC are projected to double by 2040 (2, 3). The prognosis for HCC is extremely poor in all regions of the world, and the incidence and mortality rates are roughly equivalent (4). Therefore, the most effective measure of reducing HCC-related mortality is the prevention of HCC occurrence.

The most common cause of HCC is chronic infection with hepatitis B virus (HBV) or hepatitis C virus (HCV) (2–4). By 2040, deaths from chronic viral hepatitis are projected to exceed the combined mortality rates associated with human immunodeficiency virus (HIV) infection, tuberculosis, and malaria (3). Approximately 3.9% of people worldwide, corresponding to 292 million persons, were chronically infected with HBV (CHB) in 2016, and CHB is responsible for nearly 1 million deaths each year (5, 6).

Although the universal HBV vaccination program has been successfully implemented for almost 3 decades in many countries, most HBV-related HCCs occur in unvaccinated middle-aged and elderly adults (7, 8). Therefore, considering the birth cohort effect on HBV-related HCC incidence, secondary prevention of HCC through antiviral therapy for persons already chronically infected with HBV is the primary means of reducing HBV-related deaths. In fact, it has been simulated that treating 80% of eligible individuals could reduce HBV-related deaths by 65% in the short term (5). However, less than 10% of patients with CHB in need of antiviral therapy received it in 2015 (3), and there are still serious controversies regarding the optimal time to start the antiviral treatment to prevent HCC in noncirrhotic patients with CHB.

Most patients with CHB have positive hepatitis B e antigen (HBeAg) and very high serum levels of HBV DNA (≥8 log_10_ IU/mL) at the initial phase of the infection and may eventually show a progressive decline in HBV DNA levels during the natural course of CHB infection (9–11). Thus, identifying the association between baseline HBV DNA levels and on-treatment risk of HCC in HBeAg-positive, noncirrhotic patients with CHB may provide a crucial piece of evidence to determine the optimal timing of antiviral therapy initiation.

Therefore, we aimed to evaluate the association between baseline HBV DNA levels and the risk of HCC in HBeAg-positive, noncirrhotic adult patients with CHB treated with entecavir or tenofovir through this multicenter historical cohort study.

## Results

### Patient characteristics.

**Figure 1:**
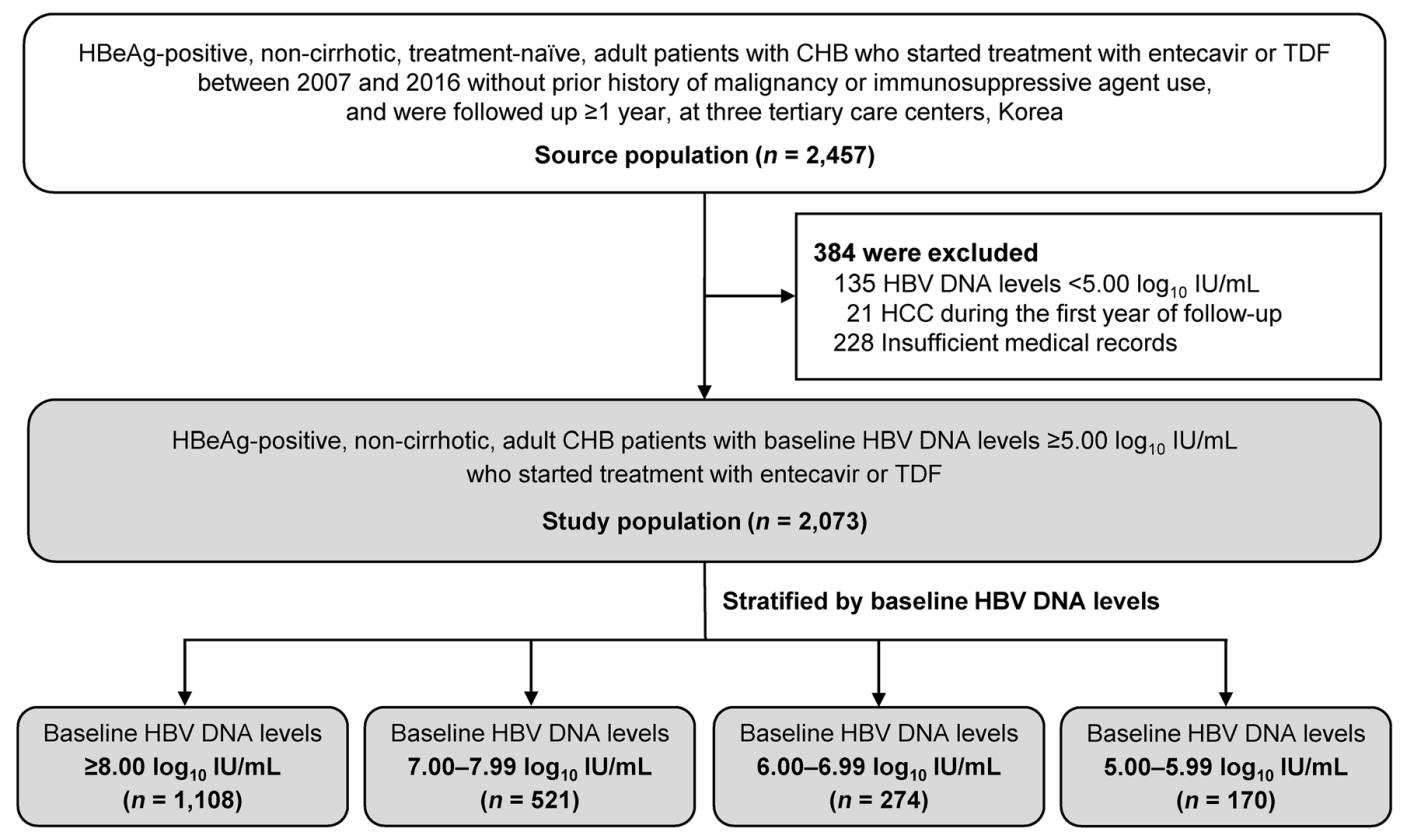
Patient flow diagram. HBeAg-positive, noncirrhotic adult patients with CHB were treated with entecavir or TDF. HBV DNA levels were measured at treatment initiation.

The study population was composed of 2073 HBeAg-positive, noncirrhotic adult patients with baseline HBV DNA levels of 5.00 log_10_ IU/mL or higher, who were initiated on treatment with either tenofovir disoproxil fumarate (TDF) or entecavir (Table 1 and Figure 1). At baseline, the mean age of the patients in the entire study was 42.1 years, 63.0% were male and 37.0% were female, and the median baseline HBV DNA level was 8.0 log_10_ IU/mL (IQR, 7.2–8.3 log_10_ IU/mL).

When categorized according to the baseline HBV DNA level, 1108 (53.4%), 521 (25.1%), 274 (13.2%), and 170 (8.2%) patients had HBV DNA levels of 8.00 log_10_ IU/mL or higher, 7.00–7.99 log_10_ IU/mL, 6.00–6.99 log_10_ IU/mL, and 5.00–5.99 log_10_ IU/mL, respectively. The baseline characteristics among the patient groups categorized by baseline HBV DNA levels were well balanced after propensity score (PS) weighting (standardized mean difference [SMD] <0.1; Table 1).

### Baseline HBV DNA levels and HCC risk.

During 5.7 years of median follow-up with continuous treatment, a total of 47 patients developed HCC (incidence rate: 0.39 per 100 person-years [PYs]). Patients who developed HCC during the study period were more likely to be older, male, have lower HBV DNA levels, alanine aminotransferase (ALT) levels, and platelet counts; but a higher fibrosis 4 (FIB-4) index and modified platelet age gender–HBV (mPAGE-B) score; and have used entecavir rather than TDF, compared with those who did not develop HCC (Supplemental Table 1; supplemental material available online with this article; https://doi.org/10.1172/JCI154833DS1).

**Figure 2:**
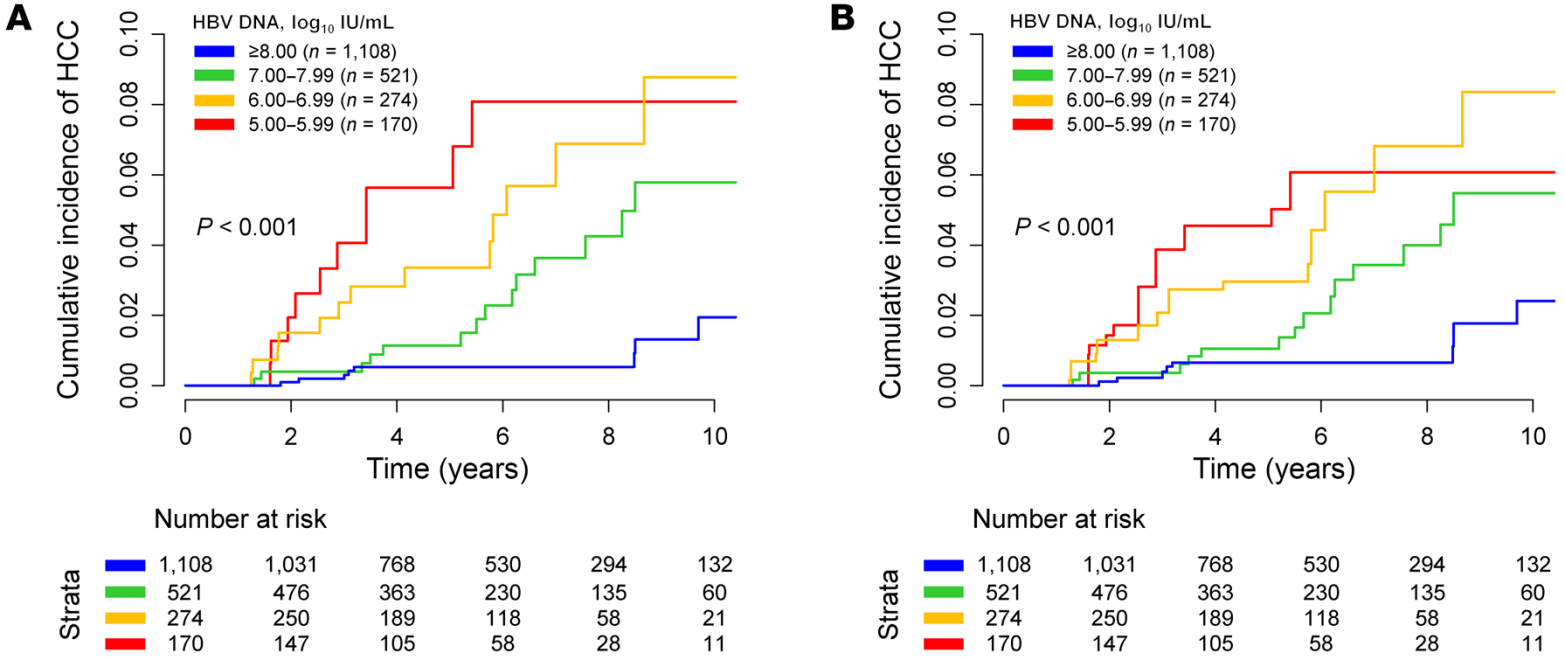
Cumulative incidence of HCC during treatment by baseline HBV DNA levels. (**A**) Unweighted analysis. (**B**) PS-weighted analysis.

**Figure 3:**
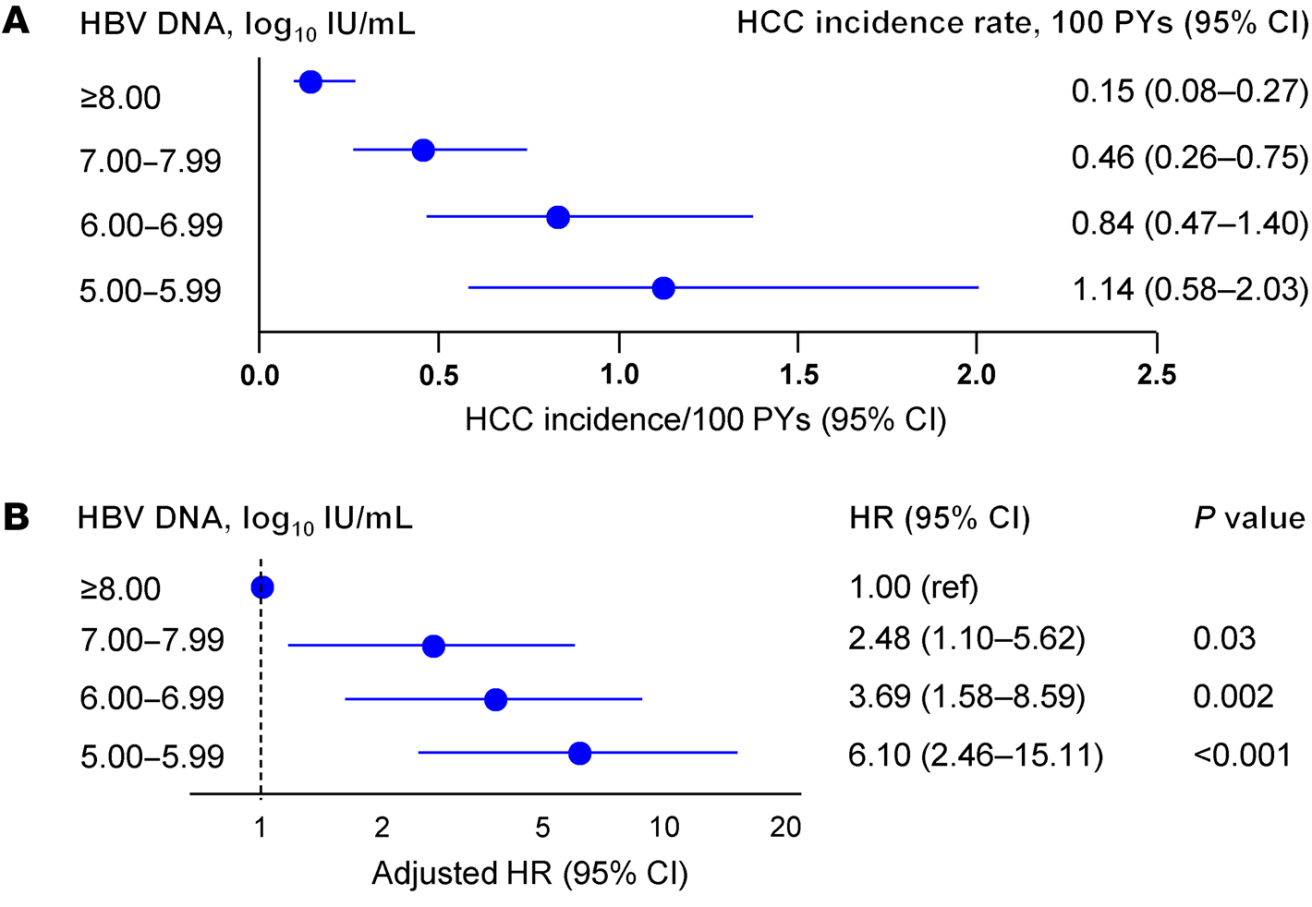
On-treatment HCC incidence rate and forest plot by baseline HBV DNA levels. (**A**) Incidence rate according to baseline HBV DNA levels. (**B**) Forest plot of multivariable-adjusted HRs according to baseline HBV DNA levels. HRs were adjusted for age, sex, platelet counts, ALT levels, and FIB-4 index. ref, reference.

Kaplan-Meier analysis showed that the risk of HCC was the lowest in those with baseline HBV DNA levels of 8.00 log_10_ IU/mL or higher, increased incrementally with decreasing baseline viral load, and the highest with HBV DNA levels of 5.00–5.99 log_10_ IU/mL (*P* < 0.001; Figure 2A). These results were consistently observed in PS-weighted analysis (*P* < 0.001; Figure 2B) and univariate Cox proportional hazard regression analysis (Table 2). The incidence rates of HCC were 0.15, 0.46, 0.84, and 1.14 per 100 PYs in individuals with baseline HBV DNA levels of 8.00 log_10_ IU/mL or higher, 7.00–7.99 log_10_ IU/mL, 6.00–6.99 log_10_ IU/mL, and 5.00–5.99 log_10_ IU/mL, respectively (Figure 3A and Supplemental Table 2).

Multivariable Cox proportional hazard regression model also showed that baseline HBV DNA levels were independently associated with the risk of HCC (Table 2 and Figure 3B). Similar to the results of the univariate analysis, the risk of HCC incrementally increased with decreasing levels of baseline HBV DNA, after adjusting for age, sex, platelet count, ALT levels, and FIB-4 index. The adjusted HRs for baseline HBV DNA levels of 7.00–7.99 log_10_ IU/mL, 6.00–6.99 log_10_ IU/mL, and 5.00–5.99 log_10_ IU/mL were 2.48 (95% CI, 1.10–5.62; *P* = 0.03), 3.69 (95% CI, 1.58–8.59; *P* = 0.002), and 6.10 (95% CI, 2.46–15.11; *P* < 0.001), respectively, with HBV DNA levels of 8.00 log_10_ IU/mL or higher as a reference. In contrast, baseline ALT levels were not significantly associated with HCC risk (Table 2).

The incremental HCC risk associated with decreasing baseline HBV DNA levels was consistently observed when baseline alpha-fetoprotein (AFP) level or antiviral treatment type (TDF or entecavir) was further adjusted in the multivariable analysis. Moreover, these similar results were reproduced after excluding patients who did not achieve viral suppression, defined as HBV DNA levels below 2000 IU/mL, at 1 year (Supplemental Table 3).

The inverse association between baseline HBV DNA levels and on-treatment risk of HCC was also consistently observed in PS-weighting and competing risk analyses with adjustment for the probability of death and liver transplantation (Supplemental Table 4).

### Sensitivity analyses.

We conducted a nested case-control study as a sensitivity analysis to identify the effect of baseline HBV DNA levels after adjusting for age, sex, and platelet counts, which may confound the risk of HCC. The control participants who did not develop HCC were randomly selected and matched with the 47 patients who developed HCC during follow-up in the entire cohort at a 1 to 4 ratio based on age, sex, platelet counts, and follow-up duration (Supplemental Table 5). The matched HCC patients had significantly lower baseline HBV DNA levels than did the controls and showed an incremental HCC risk with decreasing baseline HBV DNA levels.

We conducted another sensitivity analysis that included only patients with baseline platelet counts of 150,000/μL or higher for a more strict exclusion of those who might have advanced liver fibrosis or cirrhosis. The results were similar to those obtained in the entire cohort, with increasing risks of HCC observed with decreasing baseline HBV DNA levels from 8.00 log_10_ IU/mL or higher to 5.00–5.99 log_10_ IU/mL, by multivariable analyses with and without inclusion of the mPAGE-B score as a covariate (Supplemental Table 6 and Supplemental Figure 1).

### Stratified analyses.

We performed stratified analyses by dividing the patients according to age, mPAGE-B score, and FIB-4 index at baseline. When the patients were stratified by age at baseline (≤45 years vs. >45 years), the risk of HCC incrementally increased with decreasing baseline HBV DNA levels in both subgroups (Supplemental Table 7 and Supplemental Figure 2). The effect size of baseline HBV DNA levels was larger in younger patients compared with older patients, suggesting that baseline HBV DNA levels have a greater effect when the patients are younger.

When we stratified the patients by mPAGE-B score at baseline (<11 vs. ≥11), both groups showed an incrementally increasing on-treatment risk of HCC with decreasing baseline HBV DNA levels (Supplemental Table 8 and Supplemental Figure 3), thus indicating the additive value of baseline HBV DNA levels in predicting on-treatment HCC risk within similar risk groups by mPAGE-B score in HBeAg-positive, noncirrhotic patients.

When we stratified the patients by FIB-4 index at baseline (<2 vs. ≥2), both groups had similar results with regard to the inverse relationship between baseline HBV DNA levels and on-treatment HCC risk (Supplemental Table 9 and Supplemental Figure 4).

*PS-matching analysis: high versusmoderate viral load groups*. By penalized spline regression analysis, we found that the risk of HCC increased sharply as the baseline HBV DNA levels fell below 8.00 log_10_ IU/mL and flattened below 6.00 log_10_ IU/mL (Supplemental Figure 5). On the basis of these results, we divided the patients into 2 subgroups according to their baseline HBV DNA level: high viral load (HBV DNA levels ≥8.00 log_10_ IU/mL) and moderate viral load (5.00–7.99 log_10_ IU/mL) groups. PS matching generated 930 pairs, and all baseline characteristics were well balanced between the 2 groups (SMD <0.1; Supplemental Table 10), with the exception of baseline HBV DNA levels.

**Figure 4:**
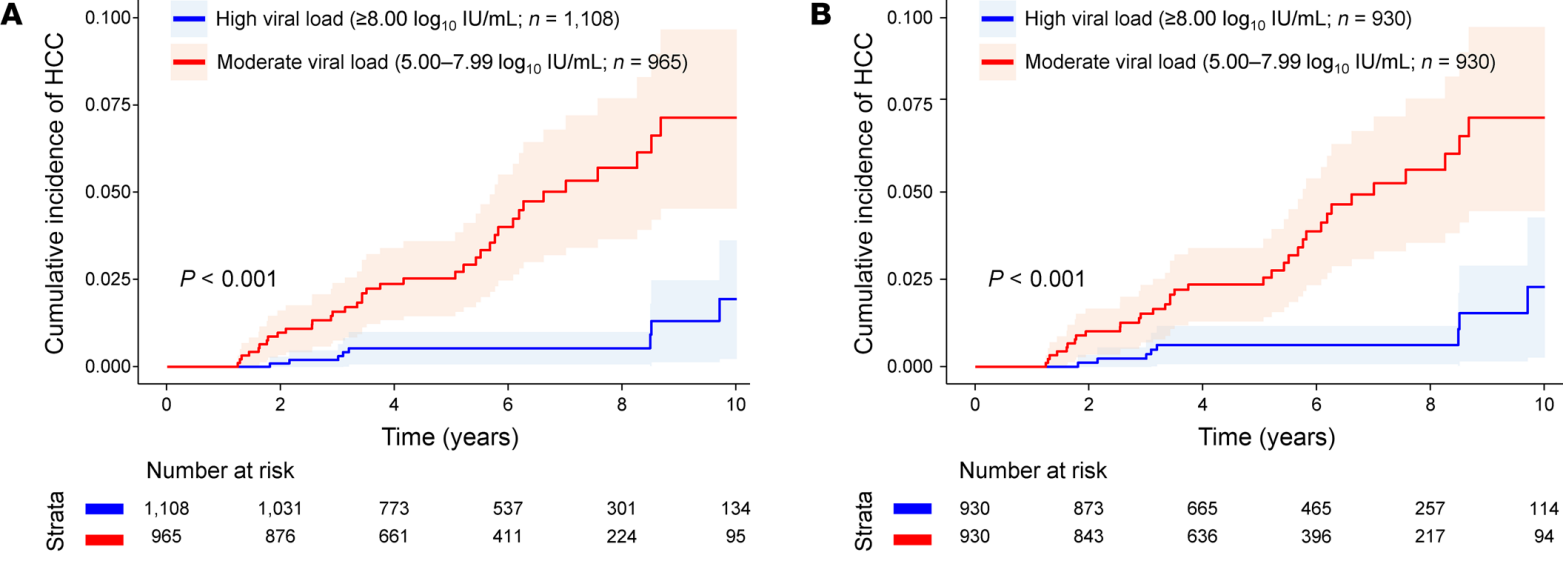
Cumulative incidence of HCC during treatment in high versus moderate viral load groups. (**A**) Entire cohort. (**B**) PS-matched cohort. Patients in the high and moderate viral load groups were defined as having baseline serum HBV DNA levels of 8.00 log_10_ IU/mL or higher or 5.00–7.99 log_10_ IU/mL, respectively.

Patients with a moderate viral load at baseline had a significantly higher HCC incidence than did those with a high viral load in both the entire cohort and the PS-matched cohort by Kaplan-Meier analysis (*P* < 0.001 for both; Figure 4). By multivariable analysis, patients in the moderate viral load group had a significantly higher risk of HCC (HR 3.48; 95% CI, 1.72–7.06; *P* < 0.001) than did those in the high viral load group, which was also consistently observed in all other analyses performed (Supplemental Table 11).

*HCC risk in the untreated versustreated cohorts*. To address whether antiviral treatment reduces the risk of HCC differently according to the baseline HBV DNA levels, we further included an untreated cohort in our analyses and compared the incidence of HCC between the untreated and treated patients after stratification by baseline HBV DNA levels (i.e., high [HBV DNA levels ≥8.00 log_10_ IU/mL] and moderate [5.00–7.99 log_10_ IU/mL] viral load groups). The untreated cohort comprised HBeAg-positive, noncirrhotic, treatment-naive adult patients with CHB who did not receive antiviral treatment because they had no significant ALT elevation during the study period (Supplemental Figure 6).

Since the baseline characteristics between the untreated and treated cohorts were significantly different, we matched the patients separately within the strata of baseline HBV DNA levels by using the PS. The variables used to derive the PS were age, sex, HBV DNA levels, and platelet counts. The PS matching generated 969 pairs for the high viral load group and 947 pairs for the moderate viral load group. After the PS matching of the patients in the untreated and treated groups, we found no significant between-group difference in baseline characteristics (SMD <0.1; Supplemental Table 12), except for liver enzyme levels and the FIB-4 index, which are the intrinsic differences between the 2 groups.

The results showed that the treated patients in the moderate viral load group had a significantly lower risk of HCC compared with the untreated patients; nonetheless, the risk was significantly higher than that of the treated patients in the high viral load group in both the multivariable and PS-matched analyses (Supplemental Table 13 and Supplemental Figure 7).

## Discussion

In this multicenter historical cohort study of 2073 HBeAg-positive, noncirrhotic adult CHB patients with baseline HBV DNA levels of 5.00 log_10_ IU/mL or higher, we found that pretreatment baseline serum HBV DNA levels had an inverse association with the risk of HCC during continuous treatment with entecavir or TDF, independent of other predictive factors. On-treatment HCC risk increased incrementally with decreasing levels of baseline HBV DNA. Compared with patients with high baseline HBV DNA levels of 8.00 log_10_ IU/mL or higher, those with HBV DNA levels of 7.00–7.99, 6.00–6.99, and 5.00–5.99 log_10_ IU/mL had a 2.48, 3.69, and 6.10 times higher adjusted risk of HCC, respectively, during continuous treatment. The inverse relationship between baseline HBV DNA levels and on-treatment HCC risk was consistently observed in unadjusted, multivariable-adjusted, PS-weighted, PS-matched, sensitivity, and competing risk analyses of the entire cohort and of the various subgroups of patients. Moreover, the HCC risk for the patients who initiated antiviral treatment with a moderate viral load was lower than that of the untreated patients with the same range of HBV DNA levels; nonetheless, it was higher than that of the patients treated who had a high viral load, indicating that the antiviral treatment could reduce the risk of HCC in the moderate viral load groups, but could not return it to the levels of the high viral load group.

The association between baseline HBV DNA levels and on-treatment HCC risk has remained unclear. Our current findings are in line with previous observations of ours and others in untreated HBeAg-positive patients, which showed that lower baseline HBV DNA levels (but above 5 log_10_ IU/mL) were associated with significantly higher risks of HCC during follow-up without treatment (12–14). However, the present multicenter cohort study provides a novel observation that baseline HBV DNA levels do have a substantial association with the risk of HCC, even during long-term treatment with potent antiviral drugs. The inverse relationship between baseline HBV DNA levels and the risk of HCC persisted for up to 10 years with continuous potent antiviral treatment in HBeAg-positive patients with CHB.

Since the fully infected liver can produce 10^9^ to 10^10^ viruses per milliliter of serum, most of the patients with HBeAg-positive CHB have very high levels (≥8 log_10_ IU/mL) of HBV DNA during the initial phase of the infection, when the hosts were truly immune tolerant (15). A low but persistent immune-mediated killing of HBV-infected hepatocytes, by the infiltration of low-level cytotoxic T lymphocytes, leads to an adaptive response of the liver over time, with the clonal emergence of HBV-resistant hepatocytes (15), resulting in a gradual decrease in HBV DNA levels, even with persistently normal ALT levels. Thus, a decreasing but considerable viral load (e.g., 5–8 log_10_ IU/mL) may indicate the progressive damage of hepatocytes, clonal hepatocyte repopulation, and a subsequent increase in the risk of HCC (16–21). Inflammatory cytokines may also have been involved in persistent inflammation, contributing to the clonal emergence (22, 23). In line with the results from these preclinical studies and our clinical findings, moderate serum HBV DNA levels (5–7 log_10_ IU/mL) were found to be a risk factor for significant hepatic inflammation in patients with CHB, despite normal ALT levels and the absence of significant fibrosis (24). Moreover, HBV DNA integration into the host’s chromosomes could be underway in HBeAg-positive patients with chronic HBV infection over a long duration, and this may further increase chromosomal instability followed by the functional loss of tumor suppressor genes or the activation of tumor-promoting genes involved in hepatocarcinogenesis (17, 18). Therefore, our data are in agreement with the findings from those in vitro studies and provide a rationale for earlier antiviral treatment based on HBV DNA levels in patients with CHB, prior to the emergence of irreversible events of hepatocarcinogenesis.

In contrast to our results, the association between baseline HBV DNA levels and on-treatment HCC risk has not been identified as a significant factor in previous studies (25–27). Such an inconsistency between those studies and our results may be due to differences in the patient population and analytical methods. Notably, we only included treatment-naive, HBeAg-positive, noncirrhotic patients with CHB who had baseline HBV DNA levels of 5.00 log_10_ IU/mL or higher. Furthermore, we analyzed the baseline HBV DNA levels as a categorical variable, considering that our previous studies found an inverse association between HBV DNA levels and HCC risk in untreated HBeAg-positive patients with CHB (12, 13).

Most practice guidelines recommend that antiviral therapies be delayed until patients show significant elevations in ALT levels or evidence of inﬂammation or fibrosis on biopsy (9–11, 28). However, if the goal of antiviral treatment is the prevention of HCC rather than the management of hepatic inflammation or fibrosis, the guidelines should be interpreted with caution, considering that HBV-associated hepatocarcinogenesis could be underway without signs of significant hepatic inflammation or fibrosis (12, 15, 29–32). Our stratified analysis based on the FIB-4 index also supports this notion that differential degrees of HBV-associated hepatocarcinogenesis according to HBV DNA levels may be actively underway during the early stages of liver fibrosis (i.e., a lower FIB-4 index). In this regard, the currently recommended timeline of antiviral treatments based on ALT levels may be a less effective means of preventing HCC, because such a strategy may cause a progressive decline in HBV DNA levels and a corresponding irreversible increase in HCC risk as a result of delayed treatment. Moreover, from the viewpoint of cost-effectiveness, the earlier treatment initiation may be a viable option, given that the currently recommended first-line anti-HBV treatments have potent long-term efficacy, high safety profiles, a high genetic barrier to resistance, and a lower cost (33).

There have been concerns regarding low virologic responses during the treatment of patients with HBeAg-positive, chronic HBV infection, who have a high viral load and persistently normal ALT levels. A previous trial demonstrated that the rate of a virologic response (HBV DNA <69 IU/mL) with TDF monotherapy was relatively lower (55%), at 192 weeks, in HBeAg-positive, immune-tolerant-phase patients with a high viral load compared with the other studies involving patients in the active phase of CHB infection (34). However, it is notable that long-term treatment with TDF in the trial was safe, with no emergence of drug-resistant HBV mutants, and most of the patients maintained low levels of HBV DNA, with only a few patients (4 of 64) having HBV DNA levels above 2000 IU/mL out to 192 weeks (34).

This study has several limitations. Because of the observational nature of the study’s design, our findings are potentially subject to bias and confounding. To counter such limitation, we applied strict inclusion criteria and performed a thorough follow-up to obtain an almost complete set of HCC incidence data. Moreover, the study cohort comprised a large number of patients, which enabled a rigorous adjustment of baseline factors. Considering that the incidence of HCC in noncirrhotic patients with CHB is relatively low, a large-sized historical cohort study may be a valid option for identifying the predictive factors for HCC in such patients (35, 36). Second, as a single-nation study, our results may not be readily generalizable in patients of other ethnicities. Specifically, our study population predominantly had genotype C HBV that was acquired through vertical transmission (37), which may be associated with a higher risk of HCC (38). Third, since liver biopsy has been very rarely performed before antiviral treatment in patients with CHB, we could not obtain an accurate fibrosis stage. Instead, we used the FIB-4 index, which was well validated in patients with CHB in evaluating the degree of liver fibrosis (39). However, unlike the detection of cirrhosis, noninvasive tests such as the FIB-4 index and transient elastography are less reliable than histological confirmation in detecting significant (≥F2) fibrosis (40). Since we excluded the patients with any evidence of cirrhosis, the impact of the FIB-4 index and imaging-based fibrosis evaluation might be attenuated.

In conclusion, we found that pretreatment baseline HBV DNA levels were inversely associated with the on-treatment risk of HCC in HBeAg-positive, noncirrhotic adult patients with CHB receiving entecavir or TDF. On-treatment HCC risk was the lowest, with high baseline HBV DNA levels (≥8.00 log_10_ IU/mL) and increased incrementally with decreasing HBV DNA levels from above 8.00 log_10_ IU/mL to 5.00 log_10_ IU/mL. Moreover, antiviral treatment significantly reduced the risk of HCC in the patients with a moderate viral load (5.00–7.99 log_10_ IU/mL), but the risk did not decrease to the level of those who initiated the treatment with a high viral load (≥8.00 log_10_ IU/mL). Therefore, early initiation of antiviral treatment when the viral load is high (≥8.00 log_10_ IU/mL) may be considered to maintain the lowest risk of HCC for HBeAg-positive, noncirrhotic adult patients with CHB.

## Methods

### Study population.

The source population (*n* = 2,457) for this study was from a historical cohort of HBeAg-positive, noncirrhotic, treatment-naive adult patients with CHB who started antiviral therapy with entecavir or TDF between January 2007 and December 2016 at 3 tertiary care centers (Asan Medical Center, Seoul National University Hospital, and Severance Hospital) in South Korea (Figure 1). Patients were excluded if they met any of the following criteria: age less than 18 years; HBeAg-negative status at treatment initiation; evidence of cirrhosis; baseline HBV DNA levels below 5 log_10_ IU/mL; prior treatment with antiviral drugs; positive for HCV, hepatitis D virus, HIV, or other hepatotropic viruses; history of malignancy or organ transplantation; treatment with immunosuppressive agents; followed for less than 1 year; diagnosis of HCC during the first year of follow-up; or insufficient medical records. Cirrhosis was defined as the presence of any of the following characteristics: coarse liver echotexture and nodular liver surface on ultrasonography; clinical features of portal hypertension (e.g., ascites, splenomegaly, and varices); and a platelet count below 100,000/μL. Consequently, a total of 2073 HBeAg-positive, noncirrhotic adult CHB patients with baseline HBV DNA levels of 5 log_10_ IU/mL or higher, who initiated treatment with entecavir or TDF, were included in the analysis as the study population (Figure 1). The interval between baseline serum HBV DNA measurement and treatment initiation was less than 1 month for all patients.

The untreated patients were enrolled between January 2000 and December 2013 at 3 tertiary care centers (Asan Medical Center, Samsung Medical Center, and Seoul National University Hospital) in South Korea (Supplemental Figure 6). All individuals in the untreated group (*n* = 2643) were HBeAg-positive, noncirrhotic, treatment-naive adult patients with CHB who did not receive antiviral treatment during the study period and had serum ALT levels lower than 2 times the upper limit of normal at baseline. The patients were excluded if they showed ALT elevation up to 2 times or more above the upper limit of normal during the first year of follow-up and were censored at 6 months after the initiation of antiviral treatment during further follow-up.

### Outcomes and follow-up evaluation.

The primary outcome was the incidence of HCC. The index date was defined as the date of the first prescription for entecavir or TDF. Life-long reimbursement was provided for the treatment, and all patients were advised to continue the treatment even after anti-HBe seroconversion until HBsAg seroclearance was achieved.

Patients were followed up with regular HCC surveillance by ultrasonography and measurement of serum AFP levels every 6 months from the index date to the date of HCC diagnosis, death, transplantation, or the last follow-up (April 30, 2020). To verify the completeness of the HCC diagnosis, the records of all patients were accessed in December 2020 through the Korean National Health Insurance Service database, which covers over 99% of the entire Korean population. This database contains a high HCC registration rate (96.5%) and highly accurate diagnoses and has been previously validated as a reliable resource for research (41).

An HCC diagnosis was based on histologic examination or typical imaging findings (nodule >1 cm with arterial hypervascularity and portal/delayed-phase washout) on either dynamic CT or MRI (42, 43). Baseline clinical information and clinical outcomes were systematically extracted from the electronic medical records of each participating center. Serum HBV DNA levels were measured using real-time PCR assay (linear dynamic detection range, 1.5 × 10^1^ to 1 × 10^9^ IU/mL). Serological markers, including HBsAg, anti-HBs, HBeAg, and anti-HBe, were detected using enzyme immunoassays (Abbott Laboratories). The HBV genotype was not determined, because greater than 98% of Korean patients with CHB have the HBV genotype C (37).

### Statistics.

All study patients who met the eligibility criteria at baseline were included in the analyses with the intention-to-treat principle. Baseline serum HBV DNA levels were analyzed as a categorical variable (5.00–5.99 log_10_ IU/mL, 6.00–6.99 log_10_ IU/mL, 7.00–7.99 log_10_ IU/mL, and ≥8.00 log_10_ IU/mL).

The baseline characteristics of the patients were compared using the χ^2^ test for categorical variables; a *t* test or 1-way ANOVA was used to compare continuous variables. The cumulative incidence of HCC was evaluated using the Kaplan-Meier method and compared using the log-rank test. Univariate and multivariable Cox proportional hazard regression models were used to determine the independent risk factors for HCC. PS-weighting and -matching analyses were used to minimize the selection bias and potential confounding variables using the following variables: age, sex, platelet count, ALT levels, FIB-4 index, and mPAGE-B score (44, 45). Competing risk analysis was also conducted for the risk of HCC after adjusting for the probability of death and liver transplantation.

A separate sensitivity analysis was conducted only in patients with baseline platelet counts of 150,000/μL or higher to further exclude those with a possibility of cirrhosis. Stratified analyses for age groups, mPAGE-B score, and FIB-4 index were conducted to account for their confounding effect on the risk of HCC. The potential relationship between baseline HBV DNA levels and the on-treatment risk of HCC was investigated across the entire cohort using penalized spline regression with 4 degrees of freedom, which allows flexible modeling of nonlinear data.

All statistical analyses were conducted using R software, version 3.6.1 (R Foundation for Statistical Computing). All analyses were based on 2-sided tests, and *P* values of less than 0.05 were considered statistically significant.

### Study approval.

The IRB at each participating center (Asan Medical Center, Seoul National University Hospital, and Severance Hospital, and Samsung Medical Center, all in Seoul, South Korea) approved this study, and the requirement for informed consent was waived, given the retrospective nature of the study.

## Author contributions

WMC and YSL designed the study. Data collection was carried out by all authors. Statistical analyses were conducted by WMC, JC, and SH, and figures were generated by WMC. All authors participated in the interpretation of data. The manuscript was written, reviewed, and edited by WMC, GAK, JC, and YSL. YSL is the guarantor of this work, and as such, has full access to all the data used in the study and takes responsibility for the integrity of the data and the accuracy of the data analysis.

## Supplementary Material

Supplemental data

ICMJE disclosure forms

## Figures and Tables

**Table 2:**
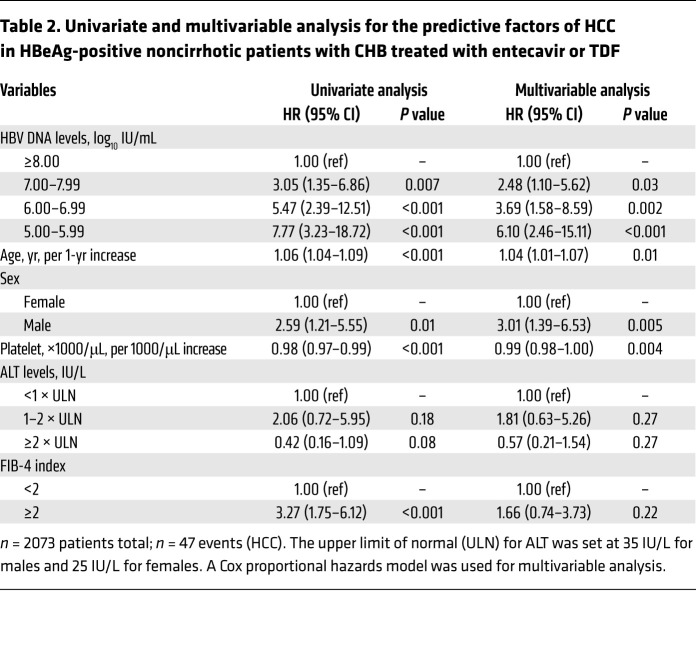
Univariate and multivariable analysis for the predictive factors of HCC in HBeAg-positive noncirrhotic patients with CHB treated with entecavir or TDF

**Table 1:**
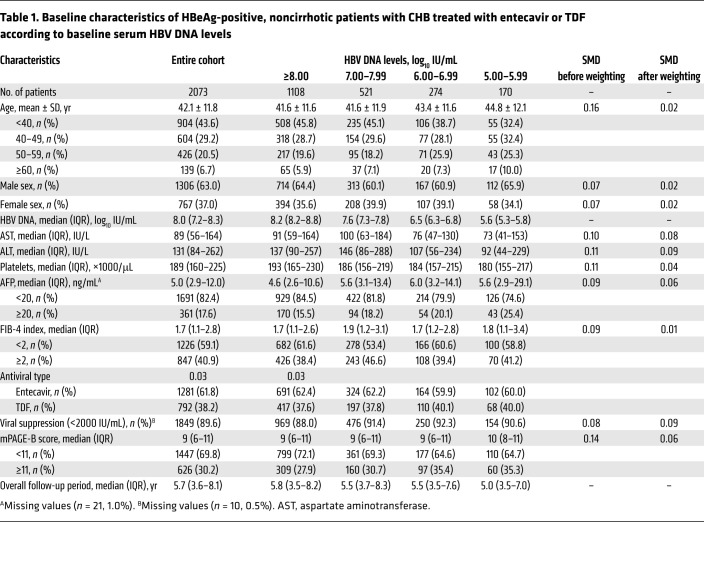
Baseline characteristics of HBeAg-positive, noncirrhotic patients with CHB treated with entecavir or TDF according to baseline serum HBV DNA levels

## Version 1 03/31/2022

In-Press Preview

## Version 2 05/16/2022

Electronic publication
