# Supplementary material for: Increasing on-treatment hepatocellular carcinoma risk with decreasing baseline viral load in HBeAg-positive chronic hepatitis B
Source: J Clin Invest. 2022 May 16;132(10):e154833. doi: 10.1172/JCI154833 (PMC9106348; doi:10.1172/JCI154833)

Online supplemental material

Increasing on-treatment hepatocellular carcinoma risk with decreasing baseline viral load in HBeAg-positive chronic hepatitis B

Won-Mook Choi, Gi-Ae Kim, Jonggi Choi, Seungbong Han, and Young-Suk Lim

Supplemental Table 1. Baseline characteristics of the patients who developed HCC and who did not during the study period.

Characteristics	No HCC	HCC	*P* value
Number	2,026	47	–
Age, mean ± SD, y	41.9±11.8	49.8±8.8	<0.001
<40, n (%)	897 (44.3)	7 (14.9)	
40-49, n (%)	592 (29.2)	12 (25.5)	
50-59, n (%)	402 (19.8)	24 (51.1)	
≥60, n (%)	135 (6.7)	4 (8.5)	
Male sex, n (%)	1,267 (62.5)	39 (83.0)	0.007
HBV DNA, median (IQR), log_{10} IU/mL	8.0 (7.2, 8.3)	7.0 (6.4, 7.9)	<0.001
≥8.00, n (%)	1,098 (54.2)	10 (21.3)	
7.00-7.99, n (%)	507 (25.0)	14 (29.7)	
6.00-6.99, n (%)	261 (12.9)	13 (27.7)	
5.00-5.99, n (%)	160 (7.9)	10 (21.3)	
AST, median (IQR), IU/L	90 (56, 166)	69 (53, 100)	0.11
ALT, median (IQR), IU/L	133 (85, 265)	82 (54, 116)	0.04
[a]AFP, median (IQR), ng/mL	5.0 (2.8, 11.8)	9.3 (5.0, 29.5)	<0.001
<20, n (%)	1,658 (82.7)	33 (70.2)	
≥20, n (%)	347 (17.3)	14 (29.8)	
Platelets, median (IQR), ×1000/μL	190 (161, 226)	160 (123, 175)	<0.001
FIB-4 index, median (IQR)	1.7 (1.1, 2.8)	2.9 (1.7, 3.8)	0.08
<2, n (%)	1,212 (59.8)	14 (29.8)	
≥2, n (%)	814 (40.2)	33 (70.2)	
Antiviral treatment type			0.02
Entecavir, n (%)	1,244 (61.4)	37 (78.7)	
TDF, n (%)	782 (38.6)	10 (21.3)	
[b]Viral suppresion (<2,000 IU/mL), n (%)	1,807 (89.6)	42 (89.4)	0.89
mPAGE-B score, median (IQR)	9 (6, 11)	12 (10, 14)	<0.001
<11, n (%)	1,434 (70.8)	13 (27.7)	
≥11, n (%)	592 (29.2)	34 (72.3)	

[a]missing values (n = 21, 1.0%); [b]missing values (n = 10, 0.5%)
Abbreviations: AFP, alpha-fetoprotein; ALT, alanine aminotransferase; AST, aspartate aminotransferase; FIB-4, fibrosis-4; HBV, hepatitis B virus; HCC, hepatocellular carcinoma; IQR, interquartile range; mPAGE-B, modified PAGE-B; PS, propensity score; SD, standard deviation; TDF, tenofovir disoproxil fumarate.

Supplemental Table 2. Incidence rates of HCC in the HBeAg-positive non-cirrhotic patients with CHB treated with entecavir or TDF.

Baseline HBV DNA levels (log_{10} IU/mL)	Person-years	No. of HCC	No. of HCC/100 person-years (95% CI)	IRR (95% CI)	*P* value
≥8.00	6,648	10	0.15 (0.08–0.27)	1.00 (ref)	–
7.00–7.99	3,069	14	0.46 (0.26–0.75)	3.03 (1.35–6.83)	0.005
6.00–6.99	1,548	13	0.84 (0.47–1.40)	5.58 (2.45–12.73)	<0.001
5.00–5.99	879	10	1.14 (0.58–2.03)	7.56 (3.15–18.17)	<0.001

CHB, chronic hepatitis B; CI, confidence interval; HCC, hepatocellular carcinoma; IRR, incidence rate ratio; TDF, tenofovir disoproxil fumarate.

Supplemental Table 3. Multivariable adjusted models for the predictive factors of HCC in the HBeAg-positive non-cirrhotic patients with CHB treated with entecavir or TDF.

Variables	Model 1		Model 2		Model 3	
	HR (95% CI)	*P* value	HR (95% CI)	*P* value	HR (95% CI)	*P* value
HBV DNA levels, log_{10} IU/mL						
≥8.00	1.00 (ref)	–	1.00 (ref)	–	1.00 (ref)	–
7.00–7.99	2.47 (1.09–5.58)	0.03	2.46 (1.09–5.56)	0.03	2.35 (0.94–5.86)	0.07
6.00–6.99	3.68 (1.58–8.55)	0.003	3.73 (1.61–8.67)	0.002	4.31 (1.75–10.63)	0.002
5.00–5.99	5.96 (2.40–14.80)	<0.001	6.07 (2.44–15.08)	<0.001	7.09 (2.71–18.59)	<0.001

Model 1 was adjusted for age, sex, platelet counts, ALT levels, FIB-4 index, and AFP levels.

Model 2 was adjusted for age, sex, platelet counts, ALT levels, FIB-4 index, AFP levels, and antiviral treatment type.

Model 3 was adjusted for age, sex, platelet counts, ALT levels, and FIB-4 index after excluding patients who did not achieve viral suppression at 1 year.

Abbreviations: AFP, alpha-fetoprotein; ALT, alanine aminotransferase; FIB-4, fibrosis-4; HBeAg, hepatitis B e antigen; HBV, hepatitis B virus; HCC, hepatocellular carcinoma; TDF, tenofovir disoproxil fumarate.

Supplemental Table 4. PS-weighted and competing risk analyses for the risk of HCC according to baseline HBV DNA levels in the entire cohort.

HBV DNA Levels, log_{10} IU/mL	HR (95% CI)	*P* value
PS-weighted analysis[a]		
≥8.00	1.00 (ref)	–
7.00–7.99	2.36 (1.04–5.36)	0.04
6.00–6.99	3.89 (1.59–9.56)	0.003
5.00–5.99	4.56 (1.73–12.01)	0.002
Competing risk analysis[b]		
≥8.00	1.00 (ref)	–
7.00–7.99	2.53 (1.12–5.75)	0.03
6.00–6.99	3.66 (1.44–9.35)	0.006
5.00–5.99	6.14 (2.46–15.30)	<0.001

[a]PS weighting variables included age, sex, platelet count, levels of alanine aminotransferase, FIB-4 index, and mPAGE-B score.

[b]Adjusted for for the probability of death and liver transplantation.

Abbreviations: FIB-4, fibrosis-4; HBV, hepatitis B virus; HCC, hepatocellular carcinoma; mPAGE-B, modified PAGE-B; PS, propensity score.

Supplemental Table 5. Association of baseline HBV DNA levels and the risk of HCC in the nested case-control study.

Characteristics	Cases (n = 47)	Controls (n = 188)	Crude OR (95% CI)	*P* value	Adjusted OR (95% CI)	*P* value
HBV DNA, median (IQR), log_{10} IU/mL	7.0 (6.4, 7.9)	7.9 (7.0, 8.2)				
≥8.00, n (%)	10 (21.2)	92 (48.9)	1.00 (ref)	–	1.00 (ref)	–
7.00-7.99, n (%)	14 (29.8)	51 (27.1)	2.53 (1.05–6.09)	0.04	2.72 (1.11–6.67)	0.03
6.00-6.99, n (%)	13 (27.7)	27 (14.4)	4.43 (1.75–11.22)	0.002	4.35 (1.66–11.39)	0.003
5.00-5.99, n (%)	10 (21.3)	18 (9.6)	5.11 (1.86–11.06)	0.002	4.87 (1.71–13.90)	0.003
Age, mean ± SD, y *(per 1-year increase)*	49.8±8.8	49.5±11.7	1.00 (0.97–1.03)	0.89	0.99 (0.95–1.02)	0.45
Male sex, n (%)	39 (83.0)	159 (84.6)	0.89 (0.38–2.10)	0.79	1.01 (0.40–2.56)	0.98
Platelets, median (IQR), x1000/μL *(per 1000/μL increase)*	160 (123, 175)	151 (129, 177)	1.00 (0.99–1.01)	0.78	1.00 (1.00–1.01)	0.32
ALT, median (IQR), IU/L	82 (54, 116)	122 (71, 238)				
<1×ULN	5 (10.6)	20 (10.6)	1.00 (ref)	–	1.00 (ref)	–
1-2×ULN	11 (23.4)	21 (11.2)	2.10 (0.62–7.11)	0.24	2.60 (0.72–9.36)	0.14
≥2×ULN	31 (66.0)	147 (78.2)	0.84 (0.29–2.42)	0.75	1.06 (0.34–3.33)	0.92
FIB-4 index, median (IQR)	2.9 (1.7, 3.8)	2.6 (1.8, 4.1)				
<2, n (%)	14 (29.8)	66 (35.1)	1.00 (ref)	–	1.00 (ref)	–
≥2, n (%)	33 (70.2)	122 (64.9)	1.28 (0.64–2.55)	0.49	2.10 (0.81–5.45)	0.13

Controls in the cohort were randomly selected and matched with the cases at a 1 to 4 ratio based on age, sex, platelet counts, and follow-up duration.
Abbreviations: ALT, alanine aminotransferase; CI, confidence interval; FIB-4, fibrosis-4; HBV, hepatitis B virus; HCC, hepatocellular carcinoma; IQR, interquartile range; OR, odds ratio; SD, standard deviation.

Supplemental Table 6. Univariate and multivariable analyses for the predictive factors of HCC in patients with baseline platelet count ≥150,000/μL.

Variables	Univariate analysis		Multivariable analysis without mPAGE-B score		Multivariable analysis with mPAGE-B score	
	HR (95% CI)	*P* value	HR (95% CI)	*P* value	HR (95% CI)	*P* value
HBV DNA levels, $\log_{10}$ IU/mL						
≥8.00	1.00 (ref)	–	1.00 (ref)	–	1.00 (ref)	–
7.00–7.99	2.61 (0.95–7.19)	0.06	2.28 (0.81–6.37)	0.12	2.30 (0.82–6.45)	0.11
6.00–6.99	5.13 (1.85–14.18)	0.002	4.24 (1.48–12.16)	0.007	4.30 (1.49–12.45)	0.007
5.00–5.99	5.27 (1.66–16.73)	0.005	4.81 (1.46–15.85)	0.01	5.05 (1.53–16.67)	0.008
Age, y, *per 1-year increase*						
	1.05 (1.02–1.08)	0.002	1.05 (1.01–1.09)	0.03	1.02 (0.97–1.08)	0.38
Sex						
Female	1.00 (ref)	–	1.00 (ref)	–	1.00 (ref)	–
Male	3.41 (1.18–9.85)	0.02	4.23 (1.44–12.38)	0.009	3.33 (1.08–10.25)	0.04
Platelet, ×1000/μL, *per 1000/μL increase*						
	0.98 (0.97–0.99)	0.006	0.99 (0.97–1.00)	0.05	0.99 (0.97–1.00)	0.08
ALT levels, IU/L						
<1×ULN	1.00 (ref)	–	1.00 (ref)	–	1.00 (ref)	–
1-2×ULN	2.93 (0.59–14.55)	0.19	2.54 (0.51–12.65)	0.26	2.48 (0.50–12.37)	0.27
≥2×ULN	0.75 (0.17–3.22)	0.70	1.22 (0.27–5.56)	0.80	1.26 (0.27–5.74)	0.77
FIB-4 index						
<2	1.00 (ref)	–	1.00 (ref)	–	1.00 (ref)	–
≥2	2.00 (0.95–4.19)	0.07	1.25 (0.49–3.15)	0.64	1.10 (0.43–2.85)	0.84
mPAGE-B score						
<11	1.00 (ref)	–	–	–	1.00 (ref)	–
≥11	4.78 (2.26–10.10)	<0.001	–	–	2.17 (0.69–6.86)	0.19

The upper limit of normal for ALT was 35 IU/L for males and 25 IU/L for females.
Abbreviations: ALT, alanine aminotransferase; FIB-4, fibrosis-4; HBV, hepatitis B virus; HCC, hepatocellular carcinoma; mPAGE-B, modified PAGE-B.

Supplemental Table 7. Stratified analysis by age (≤45 vs. >45 years) at baseline for the predictive factors of HCC.

HBV DNA levels, $\log_{10}$ IU/mL	Age ≤45 years		Age >45 years	
	HR (95% CI)	*P* value	HR (95% CI)	*P* value
Unadjusted analysis				
≥8.00	1.00 (ref)	–	1.00 (ref)	–
7.00–7.99	2.81 (0.63–12.57)	0.18	3.29 (1.25–8.65)	0.02
6.00–6.99	8.20 (1.96–34.36)	0.004	4.01 (1.44–11.14)	0.008
5.00–5.99	11.21 (2.51–50.12)	0.002	6.61 (2.20–19.87)	<0.001
Multivariable analysis[a]				
≥8.00	1.00 (ref)	–	1.00 (ref)	–
7.00–7.99	2.42 (0.54–10.88)	0.25	2.60 (0.98–6.91)	0.06
6.00–6.99	8.13 (1.92–34.45)	0.004	3.27 (1.15–9.34)	0.03
5.00–5.99	8.05 (1.74–37.24)	0.008	6.16 (1.97–19.22)	0.002
PS-weighted analysis[b]				
≥8.00	1.00 (ref)	–	1.00 (ref)	–
7.00–7.99	2.99 (0.68–13.21)	0.15	2.85 (1.08–7.52)	0.04
6.00–6.99	8.69 (2.10–36.02)	0.003	3.57 (1.22–10.44)	0.02
5.00–5.99	10.97 (2.46–48.80)	0.002	4.90 (1.66–14.49)	0.004
Competing risk analysis[a]				
≥8.00	1.00 (ref)	–	1.00 (ref)	–
7.00–7.99	2.43 (0.54–10.91)	0.25	2.69 (0.95–7.59)	0.06
6.00–6.99	8.16 (2.01–33.13)	0.003	3.36 (1.04–10.79)	0.04
5.00–5.99	8.09 (1.62–40.29)	0.01	6.28 (1.99–19.79)	0.002

[a]Adjusted for sex, platelet count, levels of alanine aminotransferase, FIB-4 index, and mPAGE-B score.
[b]PS weighting variables included sex, platelet count, levels of alanine aminotransferase, FIB-4 index, and mPAGE-B score.

Abbreviations: FIB-4, fibrosis-4; HBV, hepatitis B virus; HCC, hepatocellular carcinoma; mPAGE-B, modified PAGE-B; PS, propensity score.

Supplemental Table 8. Stratified analysis by mPAGE-B score for the predictive factors of HCC.

HBV DNA levels, log_{10} IU/mL	mPAGE-B score <11		mPAGE-B score ≥11	
	HR (95% CI)	*P* value	HR (95% CI)	*P* value
Unadjusted analysis				
≥8.00	1.00 (ref)	–	1.00 (ref)	–
7.00–7.99	4.58 (0.84–24.99)	0.08	2.42 (0.96–6.14)	0.06
6.00–6.99	8.91 (1.62–48.93)	0.01	4.11 (1.58–10.67)	0.004
5.00–5.99	10.69 (1.75–65.18)	0.01	6.56 (2.36–18.22)	<0.001
Multivariable analysis[a]				
≥8.00	1.00 (ref)	–	1.00 (ref)	–
7.00–7.99	4.76 (0.87–26.17)	0.07	2.11 (0.82–5.38)	0.12
6.00–6.99	9.75 (1.76–54.17)	0.009	3.16 (1.20–8.33)	0.02
5.00–5.99	9.85 (1.54–62.89)	0.002	5.22 (1.84–14.77)	0.002
PS-weighted analysis[b]				
≥8.00	1.00 (ref)	–	1.00 (ref)	–
7.00–7.99	4.96 (0.95–25.76)	0.06	2.08 (0.80–5.43)	0.14
6.00–6.99	8.81 (1.51–51.31)	0.02	3.41 (1.27–9.14)	0.02
5.00–5.99	10.68 (1.48–76.89)	0.02	4.95 (1.76–13.89)	0.002
Competing risk analysis[a]				
≥8.00	1.00 (ref)	–	1.00 (ref)	–
7.00–7.99	4.80 (0.89–25.97)	0.07	2.14 (0.83–5.54)	0.12
6.00–6.99	9.83 (1.91–50.67)	0.006	3.17 (1.14–8.79)	0.03
5.00–5.99	9.74 (1.32–72.12)	0.03	5.30 (1.86–15.06)	0.002

[a]Adjusted for age, sex, platelet count, levels of alanine aminotransferase, and FIB-4 index.
[b]PS weighting variables included age, sex, platelet count, levels of alanine aminotransferase, and FIB-4.
Abbreviations: FIB-4, fibrosis-4; HBV, hepatitis B virus; HCC, hepatocellular carcinoma; mPAGE-B, modified PAGE-B; PS, propensity score.

Supplemental Table 9. Stratified analysis by FIB-4 index for the predictive factors of HCC.

HBV DNA levels, log_{10} IU/mL	FIB-4 index <2		FIB-4 index ≥2	
	HR (95% CI)	*P* value	HR (95% CI)	*P* value
Unadjusted analysis				
≥8.00	1.00 (ref)	–	1.00 (ref)	–
7.00–7.99	4.94 (0.90–26.97)	0.07	2.29 (0.90–5.80)	0.08
6.00–6.99	10.77 (2.09–55.52)	0.005	4.09 (1.52–11.05)	0.005
5.00–5.99	10.84 (1.81–64.90)	0.009	8.11 (2.92–22.57)	<0.001
Multivariable analysis[a]				
≥8.00	1.00 (ref)	–	1.00 (ref)	–
7.00–7.99	5.68 (1.02–31.53)	0.05	2.10 (0.81–5.45)	0.13
6.00–6.99	12.17 (2.30–64.47)	0.003	3.46 (1.23–9.68)	0.02
5.00–5.99	10.13 (1.61–63.80)	0.01	6.99 (2.44–20.04)	<0.001
PS-weighted analysis[b]				
≥8.00	1.00 (ref)	–	1.00 (ref)	–
7.00–7.99	5.31 (0.97–28.92)	0.05	2.26 (0.89–5.74)	0.09
6.00–6.99	9.28 (1.78–48.48)	0.008	3.93 (1.39–11.07)	0.01
5.00–5.99	11.80 (1.91–72.94)	0.008	7.17 (2.61–19.71)	<0.001
Competing risk analysis[a]				
≥8.00	1.00 (ref)	–	1.00 (ref)	–
7.00–7.99	5.88 (1.15–29.97)	0.03	2.14 (0.81–5.70)	0.13
6.00–6.99	12.64 (2.56–62.44)	0.002	3.53 (1.17–10.63)	0.02
5.00–5.99	10.58 (1.64–68.10)	0.01	7.12 (2.64–19.22)	<0.001

[a]Adjusted for age, sex, platelet count, levels of alanine aminotransferase, and mPAGE-B score.
[b]PS weighting variables included age, sex, platelet count, levels of alanine aminotransferase, and mPAGE-B score.
Abbreviations: FIB-4, fibrosis-4; HBV, hepatitis B virus; HCC, hepatocellular carcinoma; mPAGE-B, modified PAGE-B; PS, propensity score.

Supplemental Table 10. Baseline characteristics of the patients with high (HBV DNA levels ≥8.00 log_{10} IU/mL) vs. moderate (5.00–7.99 log_{10} IU/mL) baseline viral load.

Characteristics	Entire cohort			PS-matched cohort		
	High viral load	Moderate viral load	SMD	High viral load	Moderate viral load	SMD
Number	1,108	965	–	930	930	–
Age, mean ± SD, y	41.6±11.6	42.7±11.9	0.09	42.1±11.7	42.4±11.6	0.03
<40, n (%)	508 (45.8)	396 (41.0)		413 (44.4)	385 (41.4)	
40-49, n (%)	318 (28.7)	286 (29.6)		265 (28.5)	281 (30.2)	
50-59, n (%)	217 (19.6)	209 (21.7)		194 (20.9)	199 (21.4)	
≥60, n (%)	65 (5.9)	74 (7.7)		58 (6.2)	65 (7.0)	
Male sex, n (%)	714 (64.4)	592 (61.3)	0.06	588 (63.2)	579 (62.3)	0.02
HBV DNA, median (IQR), log_{10} IU/mL	8.2 (8.2, 8.8)	7.1 (6.3, 7.6)	–	8.2 (8.2, 8.7)	7.1 (6.3, 7.6)	–
AST, median (IQR), IU/L	91 (59, 164)	86 (53, 165)	0.08	92 (61, 171)	86 (52, 161)	0.01
ALT, median (IQR), IU/L	137 (90, 257)	123 (77, 268)	0.04	139 (91, 268)	123 (77, 258)	0.02
[a]AFP, median (IQR), ng/mL	4.6 (2.6, 10.6)	5.6 (3.1, 14.4)	0.09	4.8 (2.6, 11.1)	5.7 (3.1, 14.2)	0.07
<20, n (%)	929 (84.5)	762 (80.0)		774 (83.9)	737 (80.3)	
≥20, n (%)	170 (15.5)	191 (20.0)		149 (16.1)	181 (19.7)	
Platelets, median (IQR), ×1000/μL	193 (165, 230)	185 (156, 217)	0.18	187 (161, 224)	186 (157, 218)	0.04
FIB-4 index, median (IQR)	1.7 (1.1, 2.6)	1.8 (1.2, 3.0)	0.15	1.8 (1.1, 2.8)	1.8 (1.1, 2.9)	0.02
<2, n (%)	682 (61.6)	544 (56.4)		541 (58.2)	537 (57.7)	
≥2, n (%)	426 (38.4)	421 (43.6)		389 (41.8)	393 (42.3)	
Antiviral type			0.03			0.06
Entecavir, n (%)	691 (62.4)	590 (61.1)		591 (63.5)	565 (60.8)	
TDF, n (%)	417 (37.6)	375 (38.9)		339 (36.5)	365 (39.2)	
[b]Viral suppresion (<2,000 IU/mL), n (%)	969 (88.0)	880 (91.5)	0.11	813 (88.0)	850 (91.7)	0.12
mPAGE-B score, median (IQR)	9 (6, 11)	9 (7, 11)	0.09	9 (6, 11)	9 (7, 11)	0.02
<11, n (%)	799 (72.1)	648 (67.2)		652 (70.1)	632 (68.0)	
≥11, n (%)	309 (27.9)	317 (32.8)		278 (29.9)	298 (32.0)	
Overall follow-up duration, median (IQR), y	5.8 (3.5, 8.2)	5.4 (3.6, 7.9)	–	6.0 (3.7, 8.3)	5.4 (3.6, 7.9)	–

High and moderate viral load were defined as baseline serum HBV DNA levels ≥8.00 log_{10} IU/mL and 5.00–7.99 log_{10} IU/mL, respectively.

[a]missing values (n = 21, 1.0%); [b]missing values (n = 10, 0.5%)

Abbreviations: ALT, alanine aminotransferase; AST, aspartate aminotransferase; FIB-4, fibrosis-4; HBV, hepatitis B virus; IQR, interquartile range; mPAGE-B, modified PAGE-B; PS, propensity score; SD, standard deviation; SMD, standardized mean difference; TDF, tenofovir disoproxil fumarate.

Supplemental Table 11. Incidence and the risk of HCC in patients with high (HBV DNA levels ≥8.00 log_{10} IU/mL) vs. moderate (5.00–7.99 log_{10} IU/mL) baseline viral load.

Groups	Person-years	No. of HCC	No. of HCC/100 person-years (95% CI)	HR (95% CI)	*P* value
Entire cohort, unadjusted analysis					
High viral load	6,648	10	0.15 (0.08–0.27)	1.00 (ref)	–
Moderate viral load	5,501	37	0.67 (0.48–0.92)	4.46 (2.22–8.99)	<0.001
Entire cohort, multivariable analysis[a]					
High viral load	–	–	–	1.00 (ref)	–
Moderate viral load	–	–	–	3.48 (1.72–7.06)	<0.001
Entire cohort, PS-weighted analysis[b]					
High viral load	–	–	–	1.00 (ref)	–
Moderate viral load	–	–	–	3.38 (1.65–6.94)	<0.001
Entire cohort, competing risk analysis[a]					
High viral load	–	–	–	1.00 (ref)	–
Moderate viral load	–	–	–	3.56 (1.74–7.31)	<0.001
PS-matched cohort[b]					
High viral load	5,664	10	0.18 (0.09–0.31)	1.00 (ref)	–
Moderate viral load	5,301	35	0.66 (0.47–0.91)	3.76 (1.86–7.59)	<0.001
PS-matched cohort[b], competing risk analysis					
High viral load	–	–	–	1.00 (ref)	–
Moderate viral load	–	–	–	3.76 (1.85–7.63)	<0.001

High and moderate viral load were defined as baseline serum HBV DNA levels ≥8.00 log_{10} IU/mL and 5.00–7.99 log_{10} IU/mL, respectively.

[a]Adjusted for age, sex, platelet count, levels of alanine aminotransferase, FIB-4 index, and mPAGE-B score.

[b]PS matching and weighting variables included age, sex, platelet count, levels of alanine aminotransferase, FIB-4 index, and mPAGE-B score.

Abbreviations: FIB-4, fibrosis-4; HBV, hepatitis B virus; HCC, hepatocellular carcinoma; mPAGE-B, modified PAGE-B; PS, propensity score.

Supplemental Table 12. Baseline characteristics of the untreated and treated HBeAg-positive non-cirrhotic patients with chronic hepatitis B before and after PS-matching.

Characteristics	Before PS matching			After PS matching[a]		
	Untreated (n=2,643)	Treated (n=2,073)	SMD	Untreated (n=1,916)	Treated (n=1,916)	SMD
Age, mean ± SD, y	39.8 ± 11.3	42.1 ± 11.8	0.20	41.7 ± 11.2	41.5 ± 11.5	-0.02
<40, n (%)	1,327 (50.2)	904 (43.6)		822 (42.9)	869 (45.4)	
40-49, n (%)	766 (29.0)	604 (29.1)		612 (31.9)	561 (29.3)	
50-59, n (%)	424 (16.0)	426 (20.5)		365 (19.1)	374 (19.5)	
≥60, n (%)	126 (4.8)	139 (6.8)		117 (6.1)	112 (5.8)	
Male sex, n (%)	1,485 (56.2)	1,306 (63.0)	0.14	1,146 (59.8)	1,181 (61.6)	0.04
HBV DNA, median (IQR), $\log_{10}$ IU/mL	8.0 (6.9, 8.3)	8.0 (7.2, 8.3)	0.14	8.0 (7.0, 8.3)	8.0 (7.1, 8.2)	0.01
High viral load (≥8.00)	1,282 (48.5)	1,108 (53.4)		969 (50.6)	969 (50.6)	
Moderate viral load (5.00 – 7.99)	1,361 (51.5)	965 (46.6)		947 (49.4)	947 (49.4)	
AST, median (IQR), IU/L	33 (25, 44)	89 (56, 164)	0.61	34 (26, 44)	88 (55, 164)	0.59
ALT, median (IQR), IU/L	36 (25, 52)	131 (84, 262)	0.85	37 (26, 53)	130 (84, 264)	0.83
Platelets, median (IQR), x1000/μL	202 (169, 239)	189 (160, 225)	-0.21	196 (164, 228)	192 (163, 228)	-0.02
FIB-4 index, median (IQR)	1.0 (0.7, 1.6)	1.7 (1.1, 2.8)	0.57	1.2 (0.7, 1.8)	1.7 (1.1, 2.7)	0.46
<2, n (%)	2,174 (82.3)	1,226 (59.1)		1,526 (79.6)	1,170 (61.1)	
≥2, n (%)	469 (17.7)	847 (40.9)		390 (20.4)	746 (38.9)	
Overall follow-up period, median (IQR), y	7.8 (4.9, 12.0)	5.7 (3.6, 8.1)	–	7.8 (4.9, 11.9)	5.6 (3.5, 8.0)	–

[a] Matching variables: Age, sex, HBV DNA levels, and platelet count.
Abbreviations: ALT, alanine aminotransferase; AST, aspartate aminotransferase; FIB-4, fibrosis-4; HBeAg, hepatitis B e antigen; HBV, hepatitis B virus; IQR, interquartile range; PS, propensity score; SMD, standardized mean difference.

Supplemental Table 13. Univariate and multivariable analysis for the predictive factors of HCC in the untreated and treated HBeAg-positive non-cirrhotic patients with CHB.

Variables	Univariate analysis		Multivariable analysis	
	HR (95% CI)	*P* value	HR (95% CI)	*P* value
**Groups stratified by treatment status and HBV DNA levels (high vs. moderate viral load)**				
Treated, high	1.00 (ref)	–	1.00 (ref)	–
Untreated, high	1.36 (0.64–2.88)	0.43	1.46 (0.64–3.31)	0.36
Treated, moderate	4.58 (2.28–9.20)	<0.001	3.78 (1.88–7.61)	<0.001
Untreated, moderate	8.61 (4.54–16.36)	<0.001	6.17 (3.03–12.58)	<0.001
**Age, y, *per 1-year increase***				
	1.07 (1.06–1.08)	<0.001	1.05 (1.04–1.07)	<0.001
**Sex**				
Female	1.00 (ref)	–	1.00 (ref)	–
Male	1.97 (1.47–2.65)	<0.001	2.06 (1.52–2.77)	<0.001
**Platelet, ×1000/μL, *per 1000/μL increase***				
	0.98 (0.98–0.98)	<0.001	0.99 (0.98–0.99)	<0.001
**ALT levels, IU/L**				
<1×ULN	1.00 (ref)	–	1.00 (ref)	–
1-2×ULN	1.99 (1.44–2.77)	<0.001	1.25 (0.90–1.74)	0.19
≥2×ULN	0.74 (0.50–1.09)	0.13	0.73 (0.45–1.16)	0.18
**FIB-4 index**				
<2	1.00 (ref)	–	1.00 (ref)	–
≥2	4.51 (3.46–5.87)	<0.001	1.33 (0.92–1.92)	0.13

Total number of patients, 4,716; number of events (HCC), 233.
The upper limit of normal for ALT was set at 35 IU/L for males and 25 IU/L for females.
Cox proportional hazards model was used for multivariable analysis.
Abbreviations: ALT, alanine aminotransferase; CHB, chronic hepatitis B; CI, confidence interval; FIB-4, fibrosis-4; HBeAg, hepatitis B e antigen; HBV, hepatitis B virus; HCC, hepatocellular carcinoma; HR, hazard ratio.

Supplemental Figure 1. On-treatment HCC incidence rate and forest plot according to baseline HBV DNA levels in patients with a baseline platelet count ≥150,000/μL.

(A) Incidence rate according to baseline HBV DNA levels

(B) Forest plot for multivariable-adjusted HRs according to baseline HBV DNA levels.

HRs were adjusted for age, sex, platelet counts, ALT levels, FIB-4 index, and mPAGE-B score. ALT, alanine aminotransferase; CI, confidence interval; FIB-4, fibrosis-4; HBV, hepatitis B virus; HCC, hepatocellular carcinoma; HR, hazard ratio; mPAGE-B, modified PAGE-B; PY, person-year.

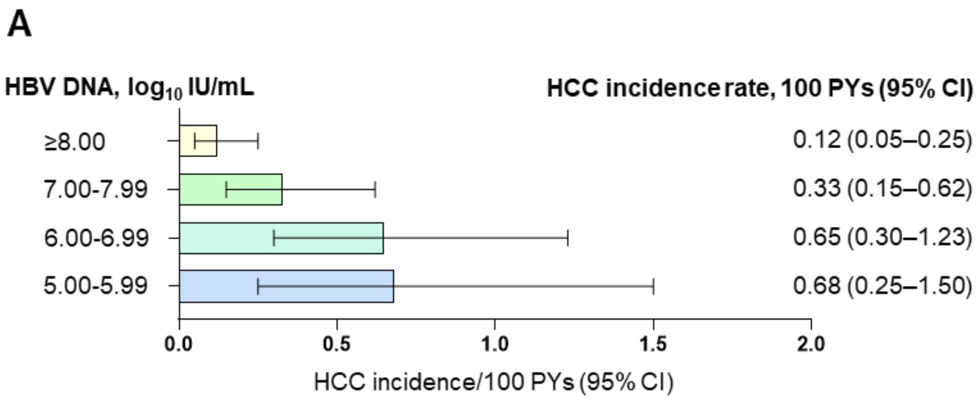

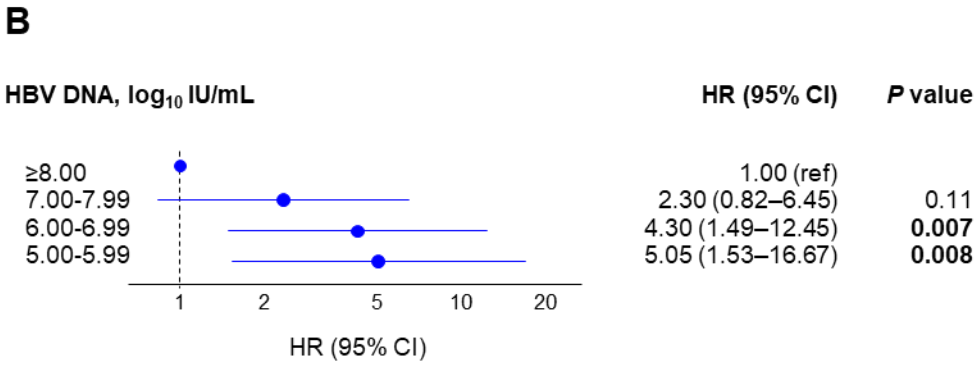

Supplemental Figure 2. Forest plots by multivariable analysis for the risk of HCC according to baseline HBV DNA levels in patients stratified by age.

(A) Patients with age ≤45 years at baseline

(B) Patients with age >45 years at baseline.

HRs were adjusted for age, sex, platelet counts, ALT levels, FIB-4 index, and mPAGE-B score. ALT, alanine aminotransferase; CI, confidence interval; FIB-4, fibrosis-4; HBV, hepatitis B virus; HCC, hepatocellular carcinoma; HR, hazard ratio; mPAGE-B, modified PAGE-B.

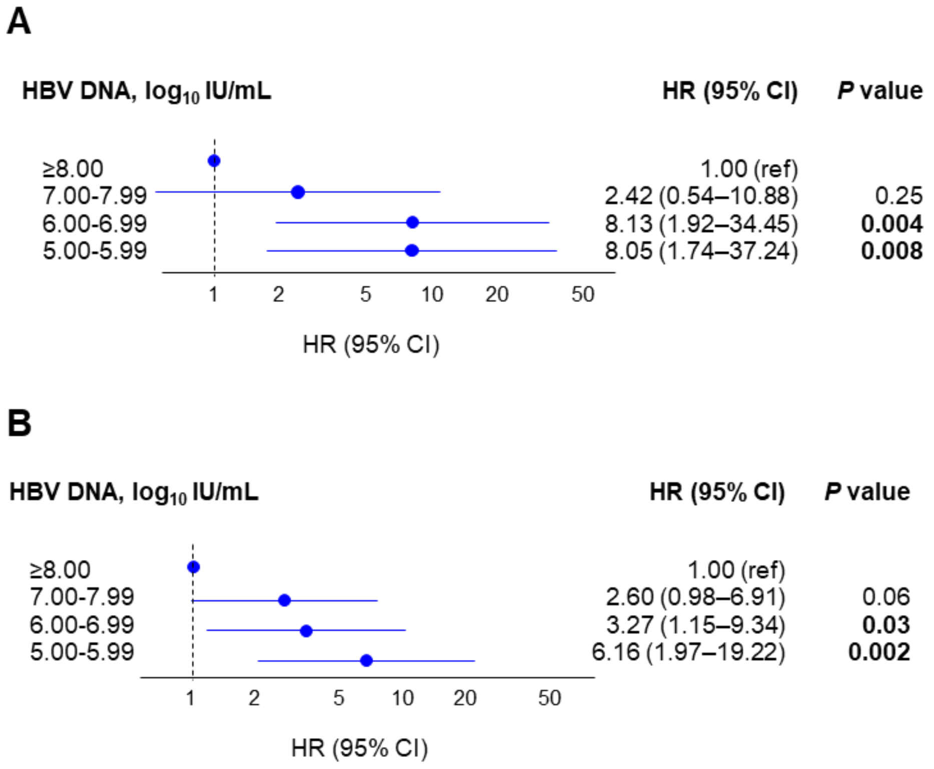

Supplemental Figure 3. Forest plots by multivariable analysis for the risk of HCC according to baseline HBV DNA levels in patients stratified by mPAGE-B score.

(A) Patients with mPAGE-B score <11 at baseline

(B) patients with mPAGE-B score ≥11 at baseline.

HRs were adjusted for age, sex, platelet counts, ALT levels, and FIB-4 index.

ALT, alanine aminotransferase; CI, confidence interval; FIB-4, fibrosis-4; HBV, hepatitis B virus; HCC, hepatocellular carcinoma; HR, hazard ratio; mPAGE-B, modified PAGE-B.

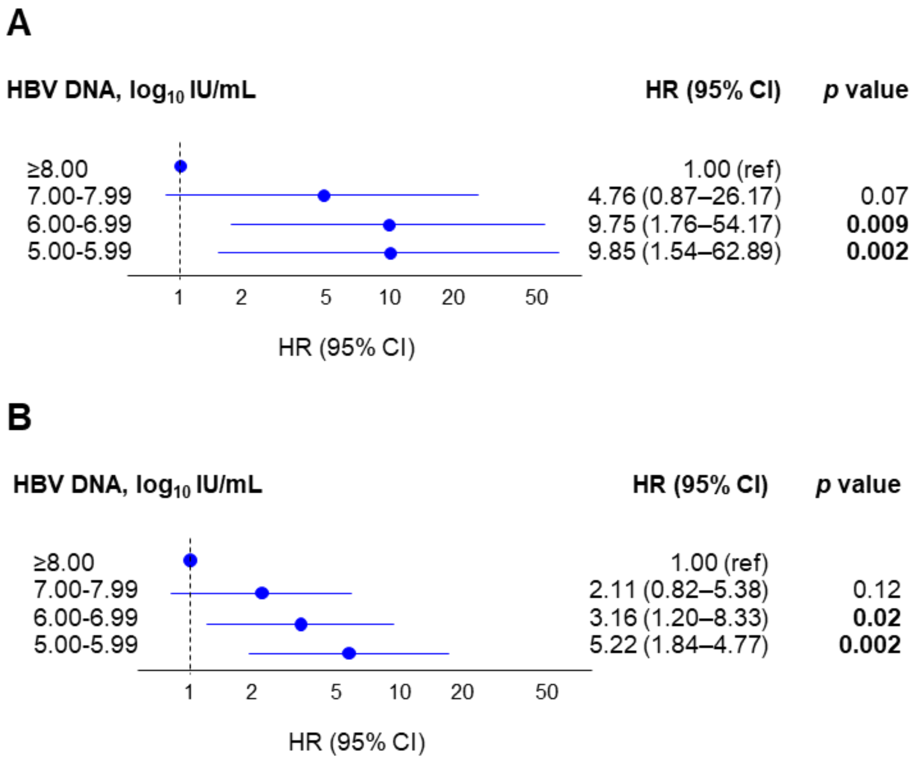

Supplemental Figure 4. Forest plots by multivariable analysis for the risk of HCC according to baseline HBV DNA levels in patients stratified by FIB-4 index.

(A) Patients with FIB-4 index <2.0 at baseline

(B) patients with FIB-4 index ≥2.0 at baseline.

HRs were adjusted for age, sex, platelet counts, ALT levels, and mPAGE-B score.

ALT, alanine aminotransferase; CI, confidence interval; FIB-4, fibrosis-4; HBV, hepatitis B virus; HCC, hepatocellular carcinoma; HR, hazard ratio; mPAGE-B, modified PAGE-B.

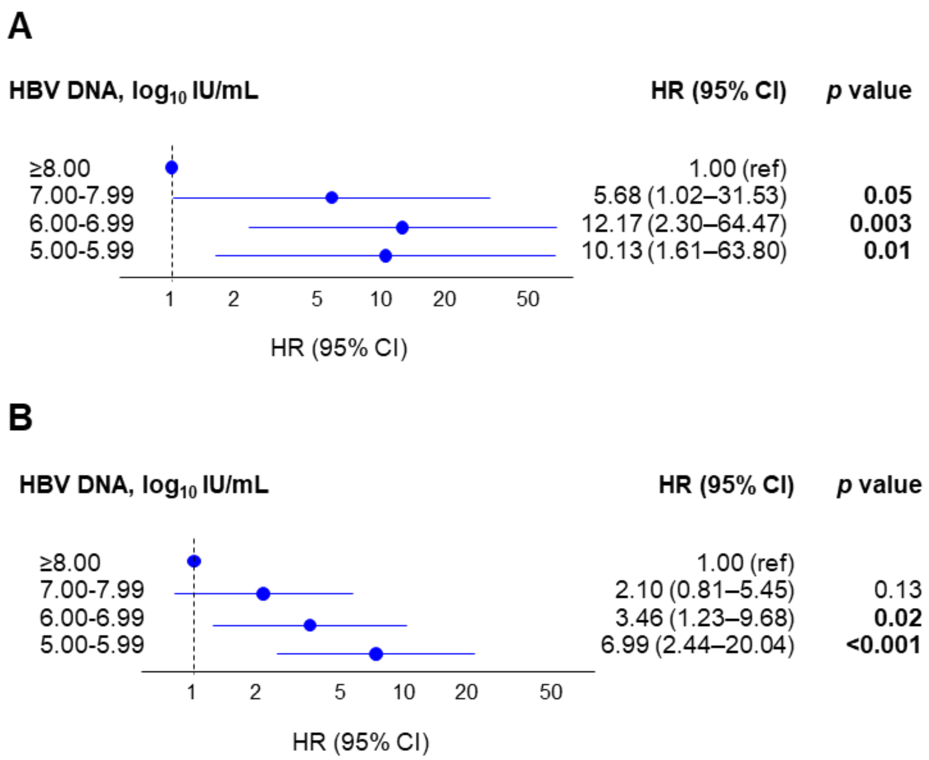

Supplemental Figure 5. Relationship between baseline HBV DNA levels and the risk of HCC.

(A) Unadjusted HR plot

(B) HR plot adjusted for age, sex, platelet count, levels of ALT, FIB-4 index, and mPAGE-B score with HBV DNA level of 8.00 $\log_{10}$ IU/mL as a reference.

The models were fitted with restricted cubic splines with 4 knots placed at the 5th, 35th, 65th, and 95th percentiles of HBV DNA levels. The black lines represent the HR and the gray zones indicate the 95% CIs.

ALT, alanine aminotransferase; CI, confidence interval; FIB-4, fibrosis-4; HBV, hepatitis B virus; HCC, hepatocellular carcinoma; HR, hazard ratio; mPAGE-B, modified PAGE-B.

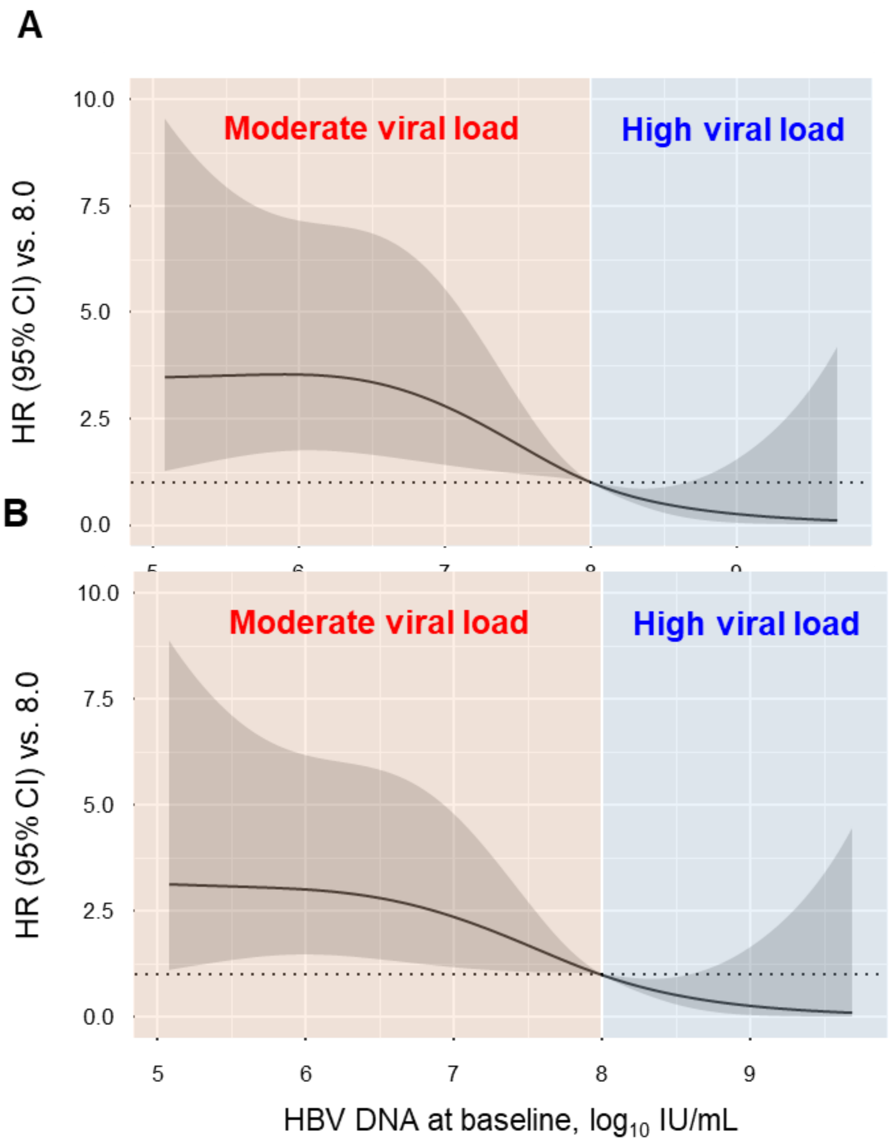

Supplemental Figure 6. Patient flow diagram for the untreated and treated HBeAg-positive non-cirrhotic patients with CHB.

ALT, alanine aminotransferase; CHB, chronic hepatitis B; HBeAg, hepatitis B e antigen; HBV, hepatitis B virus; HCC, hepatocellular carcinoma; ULN, upper limits of normal; TDF, tenofovir disoproxil fumarate.

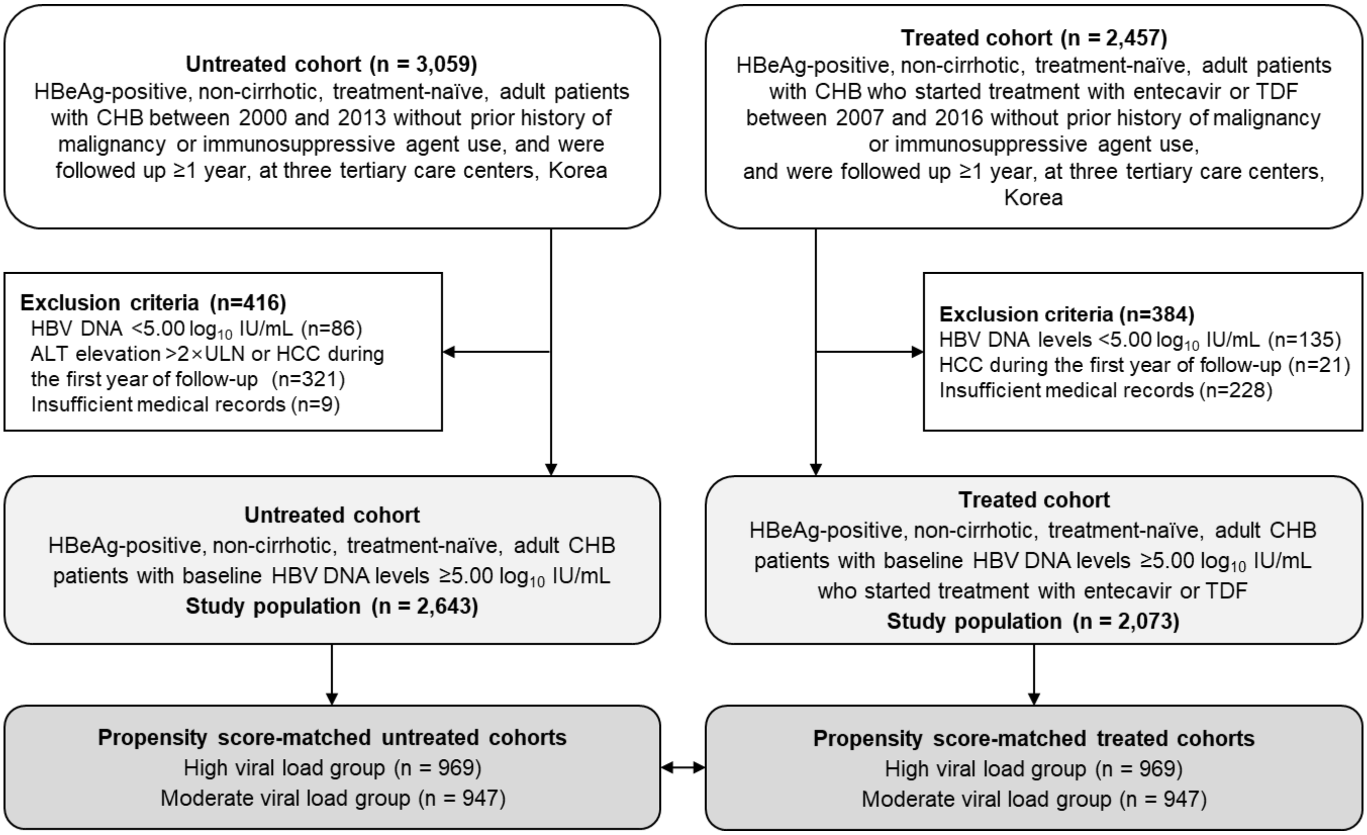

Supplemental Figure 7. Cumulative incidence of HCC in the propensity score-matched cohorts of the untreated and treated HBeAg-positive non-cirrhotic CHB patients according to baseline HBV DNA levels.

(A) Moderate viral load groups

(B) High viral load groups

(C) Adjusted HR for the risk of HCC in the entire cohorts

CI, confidence interval; HBV, hepatitis B virus; HCC, hepatocellular carcinoma; HR, hazard ratio.

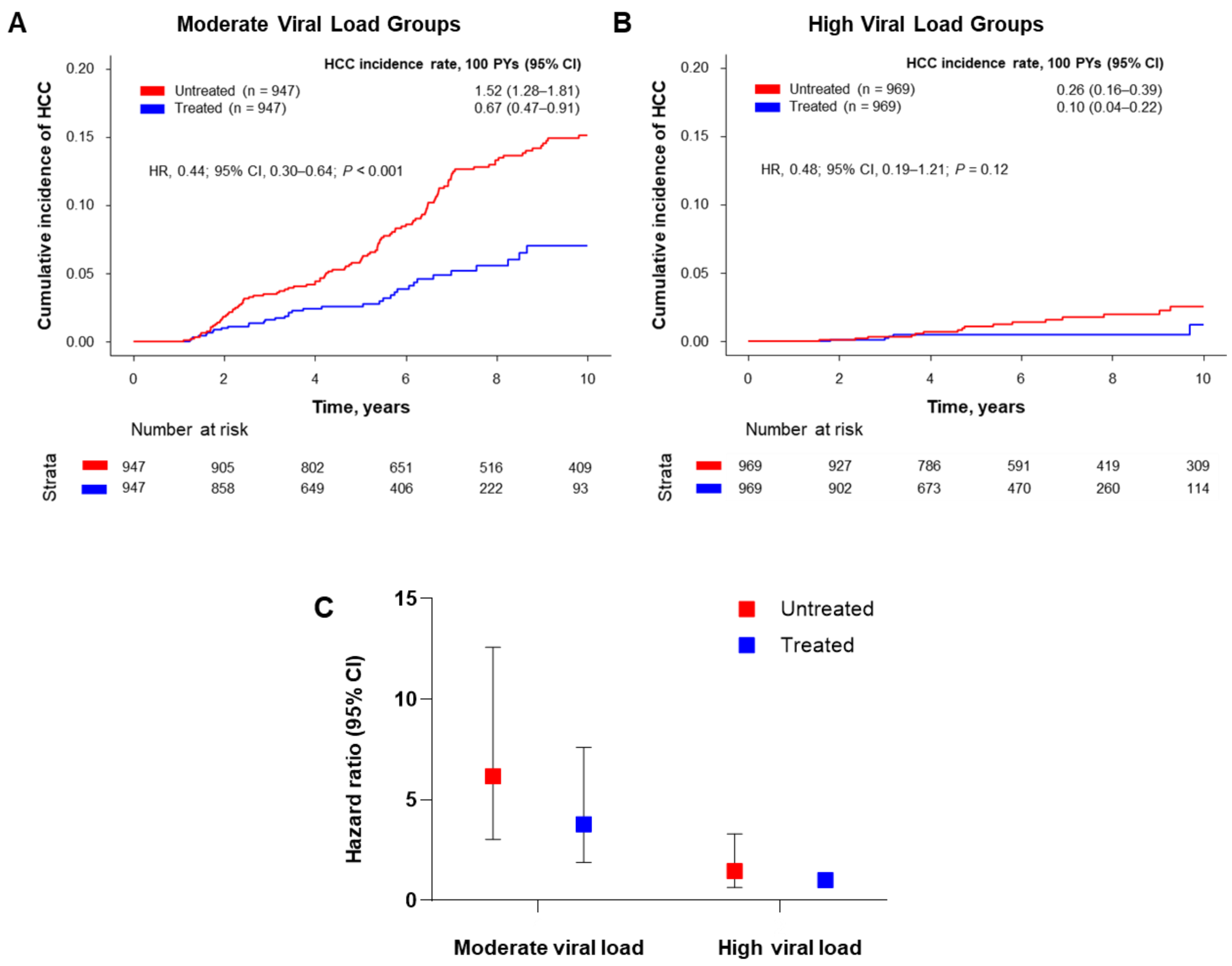

Supplement: Supplemental data [file jci-132-154833-s156.pdf]
